# Evaluating the impact of setting delineators in tunnels based on drivers’ visual characteristics

**DOI:** 10.1371/journal.pone.0225799

**Published:** 2019-12-18

**Authors:** Xueyan Han, Yang Shao, Binghong Pan, Peng Yu, Bin Li

**Affiliations:** 1 School of Highway, Chang’an University, Xi’an, Shaanxi, China; 2 Northwest Branch of China Airport Construction Group Corporation, Xi’an, Shaanxi, China; Tongii University, CHINA

## Abstract

Poor visual conditions in tunnels can easily cause traffic accidents, and it is difficult for emergency services to reach these areas. As an economical and effective visual guiding device, delineators have attracted wide attention. Based on the actual alignment of the Qinling Mountain No.1, No.2 and No.3 tunnels of the G5 Expressway in Xi’an City (Shaanxi Province, China), this paper designs a simulation experiment. Through a simulator study and a questionnaire survey, this paper discusses how delineators affect drivers’ visual characteristics (including fixation area and pupil size) in different settings and with different road alignments. Twenty-five subjects participated in this research. The results show that setting delineators in tunnels can continuously guide drivers’ vision and attract their attention to focus on the pavement. Compared with setting only pavement delineators, setting wall delineators and pavement delineators together can provide better guiding effects and ensure driving safety in both straight and curved sections. In addition, when driving in tunnels equipped with delineators, especially tunnels with both wall delineators and pavement delineators, the participants exhibited a smaller pupil diameter and lower pupil diameter change rate. In terms of the relationship between pupil size and road alignment, the results indicated that regardless of what type of delineator was used, the drivers exhibited the smallest pupil size and lowest pupil change rate when driving on the straight section compared with the curved sections.

## Introduction

In recent years, an increasing number of tunnels have been constructed in China. By the end of 2018, there were 17,738 tunnels in China, with a total length of 172,361.1 km. Compared with 2017, the number of tunnels increased by 1,509, and the length increased by 1,951 km [1]. With this increase, the problem of traffic accidents on open roads [2, 3] and in tunnels has become more serious [4–6]. Compared with accidents on open roads, traffic accidents in tunnels have serious consequences [7]. According to the Austrian highway database, the fatality rate of traffic crashes on motorways is 3.3%, but the percentage is 8.2% in tunnels [4]. The fatality rate of crashes in tunnels is thus twice as high as that outside of tunnels.

Drivers are in a dynamic environment while driving on the road. They need to pay close attention to their surroundings [8] (including other vehicles, traffic safety facilities, information boards and so on) to ensure driving safety. On different road sections, the factors that affect a driver’s driving safety are different, some studies have discussed the factors on bridges [9] and open roads [10]. On tunnel sections, when a driver is driving at high speed, the different environments inside and outside the tunnel increase the difficulty of their perception and directly affect the driving safety [11]. Especially at tunnel entrances, the dramatic change in the light intensity and the lack of traffic safety devices to lead drivers’ sight contributes to the high incidence of accidents [4, 5, 12]. To ensure safety on this section, many studies have discussed the lighting intensity design of tunnel entrances. By enhancing the illumination of the tunnel entrance, the visibility of the tunnel can be improved. Simons [13] proposed that the setting distance of the tunnel entrance illumination is related to the time when the driver adapts to low illumination. Nakamichi et al. [14] used a dynamic simulation method to study tunnel illumination and obtained a relationship model between the background brightness of the observed object and the observer’s attention time. Ragnar [15] modified the relationship between dark adaptation and light sensitivity using a dark adaptation test. However, in addition to ensuring the lighting conditions for the driver at the tunnel entrance, it is also necessary to provide effective visual guidance facilities to correctly guide the driver’s sight in this section.

Another major factor affecting tunnel safety is the monotonous environment in the tunnel. The monotonous environment encountered when driving in tunnels makes drivers anxious [16], which increases their mental demand [17]. When driving in tunnels without visual guidance facilities, drivers rely on tunnel walls to identify bends. They feel fatigued easily due to the dark environment and monotonous tunnel walls. Especially in long tunnels, the driver’s attention is gradually diminished [18]. Previous studies have found that changing the design of the tunnel’s internal facilities can mitigate this psychological problem of drivers. Through analyzing changes in the pupil diameter and heart rate, Zhao [19] suggest improving the road environment in tunnels to decrease drivers’ anxiety and mental workload. Calvi [20] proposed widening the right shoulder in tunnels to reduce the physiological and psychological stress. However, these measures can not solve the problem that drivers are prone to visual fatigue due to a lack of visual stimulation. Thus, appropriate visual stimuli are needed to increase driver’s attention and vigilance in tunnels [21].

Although the tunnel design and illumination have influences on a driver’s driving behavior, the driver’s visual attention is the most critical factor [22]. In a tunnel, a monotonous scene reduces visual attention, and drivers easily become fatigued and gradually lose their speed perception [17]. Thus, drivers in monotonous tunnels prefer to look away from the road to seek visual stimulation, which has a serious impact on driving safety [23, 24]. On open roads, rumble strips, pavement markings [25, 26] and other facilities are set to attract the attention of drivers and avoid fatigue driving. For tunnel section, many studies have discussed the influence of tunnel construction on visual characteristics. Taking the number of glances away from the road in front of the driver as an indicator, Katja [22] found that different light-colored tunnel walls can make drivers focus on forward continuity. To solve the problem of driver fatigue in long tunnels, simulator studies have been performed [27], and the results have shown that tunnel construction can affect gaze behavior. Therefore, setting an effective visual guiding device to maintain a driver’s visual focus has a significant effect on driving safety in tunnels.

Delineators can effectively attract a driver’s attention [28], improve a driver’s awareness of road alignment and effectively reduce vehicle deviation from the road in bad weather and rear-end accidents at night [29, 30]. As a function of size and spacing, four delineators appearing simultaneously in a driver’s visual field provide the best perception accuracy [31, 32], especially on a curve. Studies have also shown that the presence of delineators along the road can positively influence driver speed [33, 34]. Different delineator settings influence drivers’ behavior in tunnels differently [35–37]. For example, setting closely spaced delineators on a curve with a small radius can decrease driving speed [34]. In addition, compared with left curves, on right curves, drivers drive at higher speeds [33].

As the discussion above shows, the effect of delineators in tunnels has been discussed mostly in terms of their influence on driving speed. Few studies have focused on the effect of delineators on drivers’ visual characteristics (such as fixation area and pupil diameter). Based on the actual alignment of the Qinling Mountain No.1, No.2 and No.3 tunnels of the G5 Expressway in Xi’an City (Shaanxi Province, China), this paper designs a simulation experiment, because it is unrealistic to set different delineator configurations in an operating tunnel with a length of 18 km. Additionally, conducting this experiment in a simulator can minimize the interference of external factors and make the viewpoint information extracted for this study more valuable. This experiment needed to collect the participants’ viewpoint position data, and in order to obtain accurate data, the driver was required to maintain as fixed a head position as possible.

Through a simulator experiment and a questionnaire survey, this paper discusses how delineators influence drivers’ gaze behavior. For the simulator experiment, three scenarios with three different delineators were designed to combine three different road alignments. In the questionnaire survey, the following three questions were asked:

1. Where did you feel fatigue when driving in the three scenarios?
2. How do you evaluate the delineator settings in the tunnel?
3. Which scenario was the most comfortable for you?

The aim of this study is to evaluate how delineators affect drivers’ visual characteristics in three different settings and with three different road alignments. The remainder of this paper is organized as follows: Section 2 describes the field experiments in detail. Section 3 introduces the data collection and analysis, and an analysis of the results obtained is provided in Section 4. Last, the findings of this study are summarized in Section 5.

## Materials and methods

### Participants

To ensure the accuracy of the eye data, the drivers were required not to wear glasses while driving. In total, twenty-five drivers (8 women and 17 men) participated in the study. The mean age was 29 years. The participants’ driving experience ranged from 4 years (4 participants) to 5 years (4 participants), 7 years (10 participants), 9 years (5 participants) and 12 years (2 participants), and the total distance driven varied between 2,000 km and 300,000 km. The study was approved by the Institutional Review Board of School of Highway, Chang’an University and all participants signed forms indicating informed consent.

### Driving simulator

The Forum8 Driving Simulator was used in the experiment. This simulator simulates driving by combining three-dimensional electronic information; through a combination of virtual reality technology and a cockpit, the experimenter can realistically simulate driving in a three-dimensional scene. The visual system consists of 3 liquid crystal displays providing a 120° forward field of view.

The SMI ETGTM eye tracker consists of a head-mounted eye movement instrument, a computer workstation, a mobile recording hard disk and a built-in radio system. Its sampling rate is 120 Hz for both eyes, the tracking distance is more than 40 cm, the tracking resolution is 0.1°, the gaze positioning accuracy is 0.5°, the horizontal horizon angle is 120°, and the vertical horizontal angle is 92°.

### Driving scenario and environment

**Fig 1:**
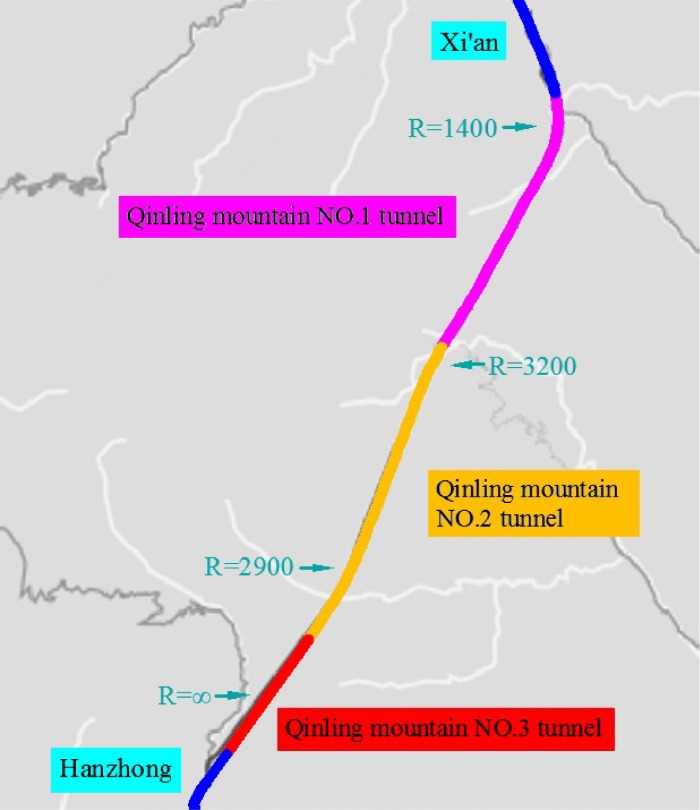
Test route alignment design. (Qinling Mountain No.1, No.2 and No.3 tunnels of the G5 Expressway).

The Qinling Mountain No.1, No.2 and No.3 tunnels of the G5 Expressway are located in Xi’an City (Shaanxi Province, China). The expressway is a two-lane road with one-tube tunnels. The total length of the three connected tunnels is 18 km. As shown in Fig 1, the driving direction is from Hanzhong to Xi’an. Within the tunnel section, there are three curves (one right and two left curves). At the beginning of the tunnel, there is a long straight section (Qinling Mountain No.3 tunnel). The overall road width is 8.5 m, the lane width is 3.75 m, the left shoulder width is 0.5 m and the right shoulder width is 0.5 m. The posted speed limit is 80 km/h.

The delineators of these three tunnels are configured differently. The Qinling Mountain No.2 tunnel is equipped with a pavement delineator, while the Qinling Mountain No.1 and No.3 tunnels have no delineators. Because of the different settings of the delineators, the gaze behavior of drivers varies greatly. To explore how delineators affect drivers’ gaze behavior in a tunnel, three different delineator post configurations were designed based on the actual alignments of the Qinling Mountain No.1, No.2 and No.3 tunnels.

The tunnel was equipped with three different delineator post configurations. Therefore, each participant was asked to drive in three scenarios:

- Scenario A, in which wall delineators were placed 7 m [38] above the pavement on the sidewall, and pavement delineators were placed on the edge marking.
- Scenario B, in which pavement delineators were placed on the edge marking.
- Scenario C, in which there were no delineators.

**Fig 2:**
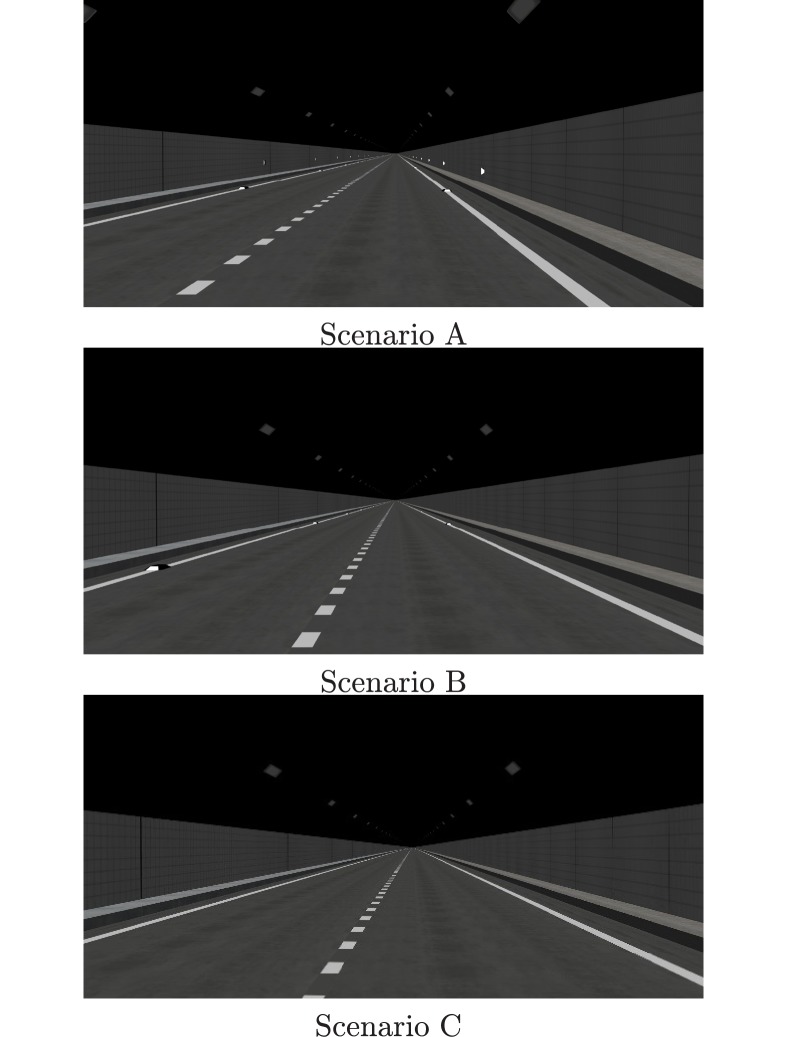
Delineator setting position in three scenarios on a straight section.

**Fig 3:**
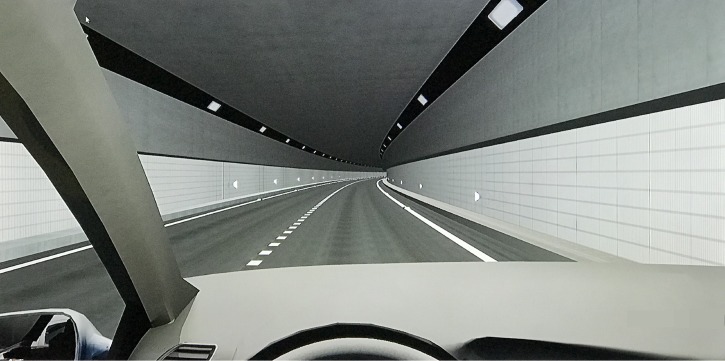
Simulation effect in the simulator. This picture is the simulation effect shown on the middle liquid crystal display when participants drove in scenario A.

The spacing was 18 m between right wall delineators, 20 m between left wall delineators, 21 m between right pavement delineators, and 22 m between left pavement delineators [39]. For example, on the straight section, the delineator setting positions in three scenarios are shown in Fig 2, and the real effect in the driving simulator when participants drove on a right curve in scenario A is shown in Fig 3.

### Experimental design

There were three delineator settings (both wall delineator and pavement delineator, pavement delineator only and no delineator) combined with three tunnel alignments (straight, left curve and right curve). The two left curve radii were 2900 m and 1400 m, and the right curve radius was 3200 m. To ensure the comparability of the data in the curve sections to the greatest extent possible, data analysis was performed on the left curve with a radius of 2900 m and the right curve with a radius of 3200 m.

The visual characteristic indicators were the participants’ fixation area and pupil diameter. To ensure the comparability of these indicators, the participants were told to drive along the right lane and, during the driving process, to identify the position where they felt fatigue.

After driving, the participants completed a questionnaire that contained three questions:

1. Where did you feel fatigue when driving in the three scenarios?
2. How do you evaluate the delineator settings in the tunnel?
3. Which scenario was the most comfortable for you?

For the first question, the result was recorded by a recorder. During the trial, the participants were asked to identify the position where they felt fatigue. Once they felt tired, they needed to tell the recorder “I feel tired now”, and then the recorder recorded the kilometers at that moment. For the second question, the ratings ranged from 0 (representing “no visual guidance function”) to 10 (representing “extremely good visual guidance function”). For the third question, participants chose which of scenario A, scenario B and scenario C were the most comfortable.

### Procedure

Because each participant was to drive in three scenarios (which took approximately 30 minutes), to save time, they were divided into 12 groups. There were two participants in each group except for the last group, which had three. To avoid the visual fatigue caused by long driving, participants could not complete all three scenarios at one time, and each participant completed the three scenarios alternately. The order of the scenarios was random to avoid bias between the trials.

Before the experiment started, the participants put on the eye tracker. They were asked to adjust their body to a comfortable sitting position and keep their heads as still as possible during the experiment. Then, they were told the design of the road and performed a 5-minute driving test in the simulator to familiarize themselves with the operating system. In addition, the participants were instructed to drive as usual, and the speed limit was 80 km/h (the driving speed of the subjects was between 75 km/h and 80 km/h, which is close to the speed limit, so this paper does not consider the influence of speed). During the trial, the participants were asked to identify the position where they felt fatigue. When the participants finished the three scenarios, they were required to evaluate the impact of the delineator settings in the tunnel.

## Analysis and results

### Heat map

Of the 25 participants, one oversped during the trials, and the eye tracker could not obtain fixation for two other participants. Therefore, the useful data came from the remaining 22 participants. The eye movement data in each trial were recorded automatically by the eye tracker and analyzed using the BeGaze 3.5 software package matched to the eye tracker.

**Fig 4:**
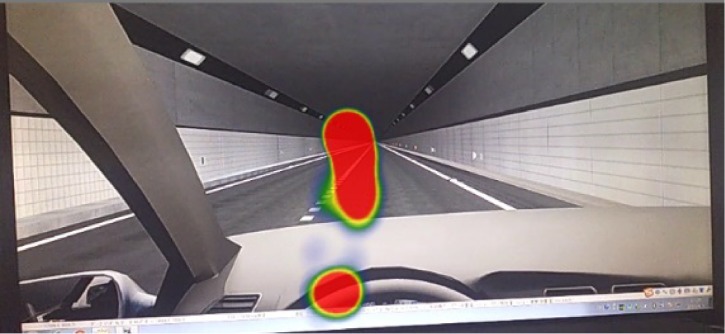
The heat map of one participant on the straight section.

Many researchers have used heat maps to analyze eye movement data [40–42], and BeGaze 3.5 can draw a heat map based on the eye movement data (fixation count and fixation time). In the heat map, the red area represents the longest fixation time, followed by the yellow area and the green area. The fixation heat map of the straight section of scenario A is shown in Fig 4. As the figure shows, this participant paid more attention to the left than the right in the tunnel ahead, and he frequently fixated on the steering wheel, meaning he cared about whether the car was speeding.

**Fig 5:**
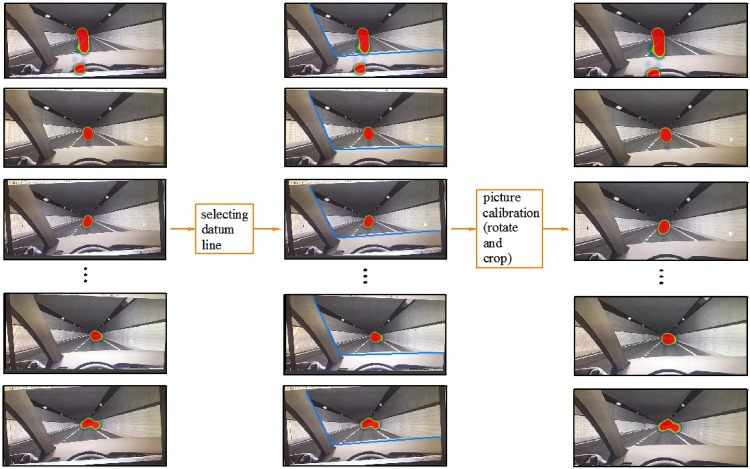
Process of calibrating images.

Because of the different positions of the participants’ heads, the angle of the participants’ views, which was recorded by the eye tracker, differed. Before analyzing these data, image calibration was required. As shown in Fig 5, the original images had different sizes and angles. Therefore, it was necessary to select a datum line to adjust these images to the same angle. When the participants sat in the simulator, the same car body contour appeared in the liquid crystal display. Therefore, the car body contour (the blue lines in Fig 5b) was chosen as the datum line. The images were rotated with the intersection of two blue lines as the datum point such that the two blue lines completely coincided. Then, the redundant edges of the images were cropped to make each image the same size, and the analyzed images were obtained (Fig 5c).

**Fig 6:**
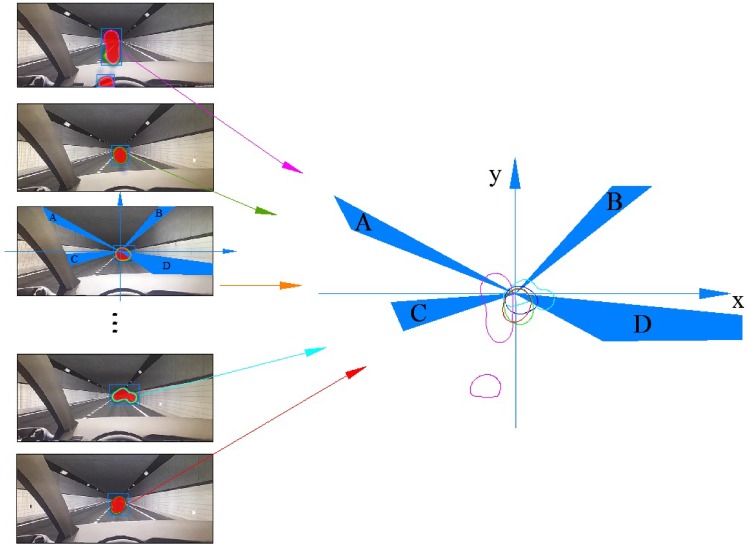
Results of extracting fixation areas.

To analyze the participants’ viewpoint position, their fixation areas in the heat map were extracted into the same coordinate system. As shown in Fig 6, the coordinate system was established with the intersection point of the boundary between the upper and lower walls as the origin. The lamp areas in the tunnel are A and B, and the delineator area are C and D. The area between C and D is pavement. The fixation areas from each heat map were drawn and extracted into the same coordinate system. Different color contours represent different participants’ fixation areas.

**Fig 7:**
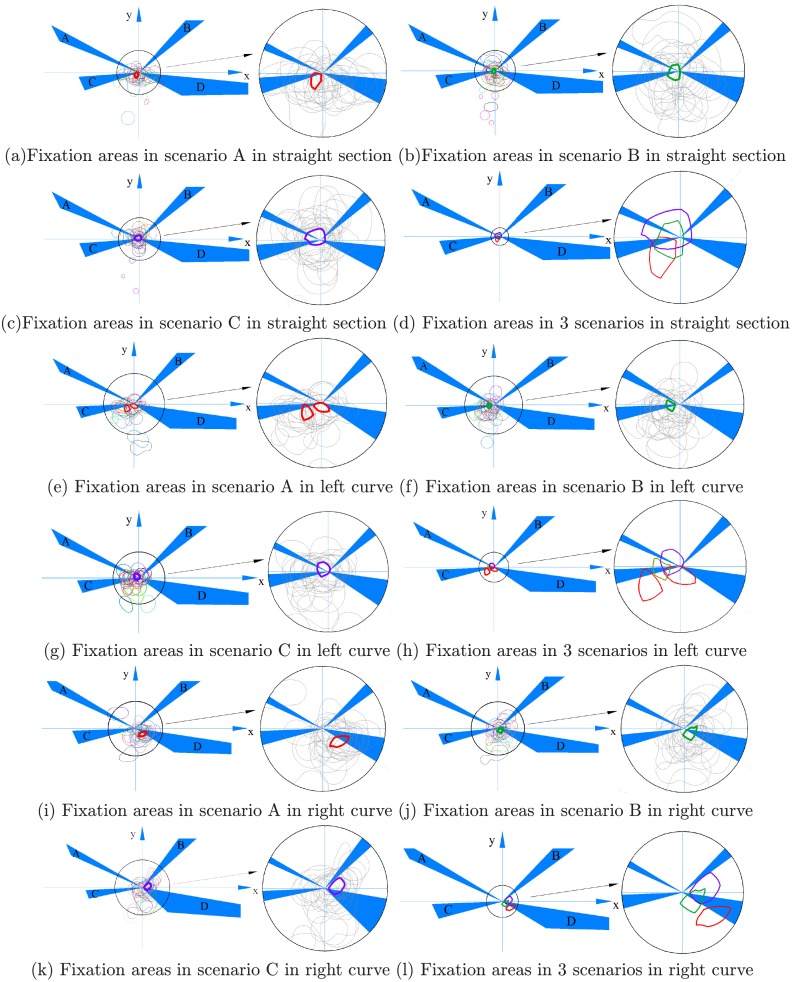
Fixation areas. In each small picture, the right part is the enlarged image in the circle on the left. In the enlarged image, to highlight the common area, only the common areas are circled with color outlines (the common areas in scenario A are circled with red outlines, the common areas in scenario B are circled with green outlines, and the common areas in scenario C are circled with purple outlines), and each participant’s fixation area is represented by a grey outline.

The extraction of the twenty-two participants’ fixation areas is shown in Fig 7. To study the characteristics of the driver’s fixation area, the common area when driving in different scenarios in the tunnel is circled. When driving in scenario A, seventeen participants saw the area that is circled in bold red on the straight section (Fig 7a). Eleven participants watched the left bold red area, and ten participants watched the right bold red area in the left curve section (Fig 7e). Fifteen participants saw the bold red area in the right curve section (Fig 7i), indicating that the majority of the participants fixated on the bold red area in scenario A during this trial. When they drove in scenario B, fourteen participants saw the area that is circled in bold green in the straight section and left curve section (Fig 7b and 7f), and seventeen participants saw the bold green area in the right curve section (Fig 7j). In scenario C, sixteen participants saw the area marked in bold purple in the straight section (Fig 7c), fifteen participants saw the bold purple area in the left curve section (Fig 7g), and thirteen participants saw the bold purple area in the right curve section (Fig 7k). The areas circled by these three bold lines were placed in the same coordinate system (Fig 7d, 7h and 7l). As shown in Fig 7d, Fig 7h and 7l, in scenario A, the common fixation area was mainly on the pavement (87% in the straight section, 89% and 87% in the left curve, and 67% in the right curve). In scenario B, approximately half of the common fixation area was on the pavement (48% in the straight section, 36% in the left curve, and 57% in the right curve), and in scenario C, a few parts of the common fixation area were on the pavement (15% in the straight section, 16% in the left curve, and 2% in the right curve). Based on the above analysis, the results showed that setting delineators in a tunnel can make the majority of drivers focus on the pavement. Because long glances away from the road are adverse to driver safety [43], the presence of delineators can increase driving safety in tunnels. The results also show that compared with scenario B, driving in scenario A attracted the participants’ attention to the pavement to the greatest extent possible. Meanwhile, according to question 2 of the questionnaire (the results are reported in Table 1), all of the participants evaluated the delineator settings positively for driving in the tunnel. In addition, according to question 3 of the questionnaire (the results are reported in Table 2), 82% of the participants thought that setting wall and pavement delineators together guided their sight better, while the other 8% thought that driving in the scenario with only pavement delineators was more comfortable.

**Table 1:** Ratings of subjective evaluation of the effect of the delineator.

Ratings	6	7	8	9	10
Number of participant	3	4	8	5	2

**Table 2:** Result of subjective choice about the most comfortable scenario.

Delineator post configuration	Scenario A	Scenario B	Scenario C
Number of participant	18	4	0

Fig 7a, 7e and 7i show that most drivers rely on wall delineators and pavement delineators to identify the roadway before them. A combination of wall delineators and pavement delineators can clearly show the edges of the roadway and the forward roadway. Thus, when driving in scenario A, the participants’ fixation areas ware mainly on the pavement and on area A and area B. When the drivers’ visual common area, shown in Fig 7e, ware analyzed, the results showed that because of different gaze habits, some drivers looked at the inner delineators in the curve section, while others preferred to look at the outer delineators. In scenario B, as shown in Fig 7b, 7f and 7j, some drivers identified the direction of the road by the pavement delineators, and others identified the direction by the lamp at the top of the tunnel. This choice also depends on the driver’s visual habits. When driving in scenario C, many drivers focused on the lamps to identify the direction of the road (Fig 7c, 7g and 7k). It seems that compared with linear structures (the edges of the roadway), the participants preferred to rely on larger structures (wall delineators, pavement delineators and lamps) to guide their viewpoints in the tunnel. The participants stated that larger structures improved the visibility of both sides of the road in the tunnel and reduced their sense of the distance of the forward road, while linear structures made the road look far away and monotonous, which made them feel easily fatigued. According to the result of the first question in questionnaire, 90% felt fatigue when they drove about 11 km in scenario A, 80% felt fatigue when they drove about 7 km in scenario B, and 92% felt fatigue when they drove about 5 km in scenario C.

### Pupil diameter

Drivers can adjust their pupil dilation to adapt to changes in the road environment. The change in pupil diameter is an important physiological and psychological index reflecting the driver’s recognition process. Reasonable sight guidance facilities will make the driver’s pupil diameter change less, make the driver more comfortable in the process of driving, and make the work load intensity smaller, which is conducive to driving safety [44]. Many studies have recognized that pupil dilation represents the arousal aspect of emotion [45, 46]. Pupil dilation is also influenced by other factors [47, 48]. In this research, except for the delineator settings, the environmental conditions were the same, so we can assume that changes in pupil diameter were affected only by the delineator setting.

**Fig 8:**
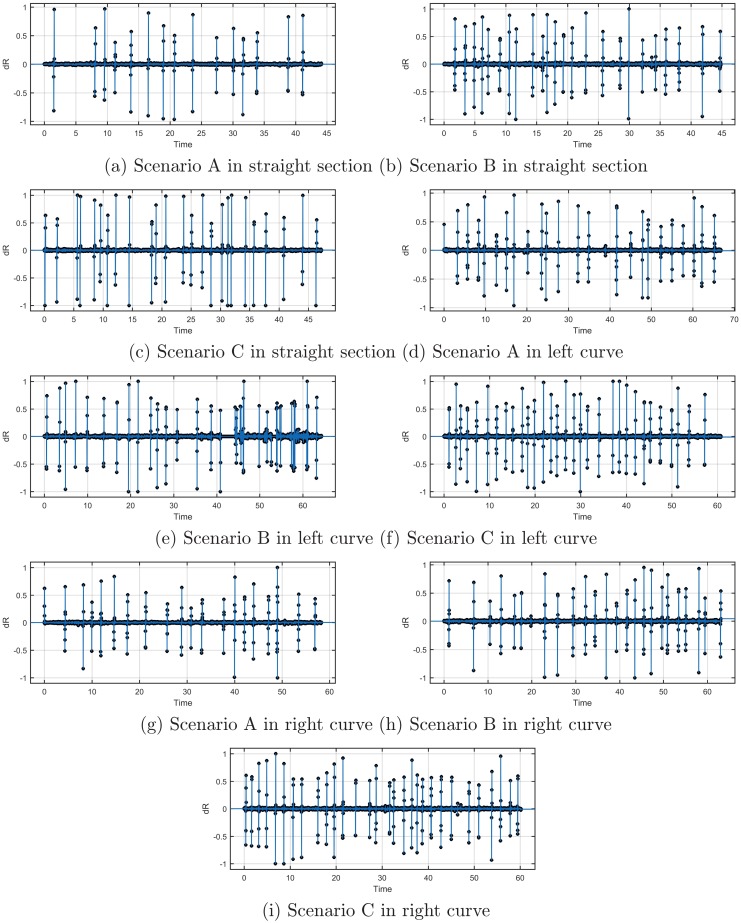
Fluctuation frequency of participants’ pupil diameter.

In this test, one of the 22 participants had incorrect pupil diameter data, so the remaining 21 were used for the analysis. The relationship between pupil diameter change and time was fitted with the interpolation method in MATLAB. The results are shown in Fig 8. When driving in scenario A, the fluctuation frequency of changes in the participants’ pupil diameter was significantly less than those in scenario B and scenario C. When they drove in scenario C, the pupil diameter fluctuation rate was the greatest. Therefore, setting delineators in a tunnel can and improve drivers’ visual adaptability and better guide their vision.

**Fig 9:**
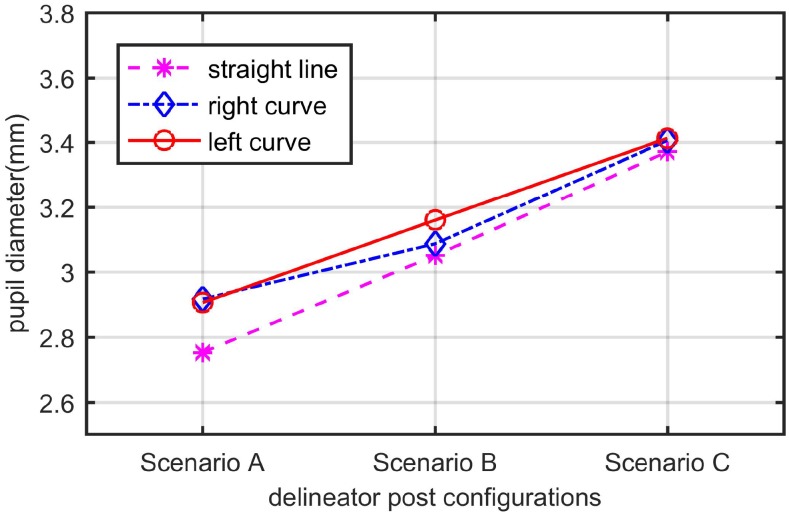
Results of extracting fixation areas.

The pupil diameter of the 21 participants in the straight section, right curve and left curve was tested by the normality test. As shown in Table 3, the distribution of these data was normal. A univariate analysis of variance (ANOVA) was used to test whether the different delineator post configurations had a significant effect on the participants’ pupil diameter changes. The dependent variable was the mean pupil diameter, and the independent variables were the three types of delineator post configurations (scenario A, scenario B and scenario C). The results showed that the delineator post configuration had a significant effect on the drivers’ pupil diameter in the straight section (F(2,60) = 12.037, P<0.001), right curve (F(2,60) = 5.845, P = 0.005) and left curve (F(2,60) = 6.927, P = 0.002). As shown in Fig 9, in the straight section, the participants’ mean pupil diameter was the largest in scenario C (3.371 mm), followed by scenario B (3.051 mm) and scenario A (2.753 mm). The same results were found for the right curve (3.406 mm in scenario C, 3.087 mm in scenario B and 2.917 mm in scenario A) and the left curve (3.413 mm in scenario C, 3.160 mm in scenario B and 2.906 mm in scenario A). In addition, the pupil diameter was the smallest when the participants drove on the straight section. There was no significant difference in pupil diameter between scenario A and scenario C when the participants drove on the left curve and right curve. However, in scenario B, the pupil diameter of the drivers was significantly larger on the left curve than on the right curve.

**Table 3:** Normality test of pupil dilation.

Variable	Group	Mean	Statistical	df	Sig.
straight	Scenario A	2.753	0.941	21	0.224
	Scenario B	3.051	0.966	21	0.643
	Scenario C	3.371	0.952	21	0.369
right curve	Scenario A	2.917	0.938	21	0.197
	Scenario B	3.087	0.964	21	0.596
	Scenario C	3.406	0.989	21	0.995
left curve	Scenario A	2.906	0.943	21	0.252
	Scenario B	3.160	0.980	21	0.925
	Scenario C	3.413	0.958	21	0.480

## Discussion

Setting delineators in a tunnel can significantly affect drivers’ visual characteristics. Delineators can make drivers focus on the road and reduce the change in pupil diameter, which benefits driver safety in tunnels.

Different methods of setting delineators have different effects on drivers’ sight guidance and thus on their visual characteristics. It is advantageous for driving safety to set both wall delineators and pavement delineators rather than only pavement delineators. When driving in scenario B, in addition to paying attention to pavement delineators, the drivers also noted the lamps at the top of the tunnel to identify the forward roadway. In scenario A, the edges of the roadway and the forward roadway were clearly shown, which made the drivers focus mainly on the pavement.

Changes in pupil diameter were frequent in scenario C. The drivers stated that in this scenario, they became fatigued easily, felt nervous and needed to concentrate. However, in scenario A and scenario B, and especially in scenario A, the drivers had a lower mental workload and felt more comfortable. Thus, setting delineators can improve drivers’ visual adaptability.

The drivers’ pupil diameter was larger on the right curve and left curve than on the straight section. Compared with the left curve and right curve, on the straight section, the driving environment was simpler. Affected by geometry, drivers will be more nervous when driving on a left curve or right curve owing to the blind areas before them. Therefore, the pupil diameter is larger than on the straight section.

Setting both wall delineators and pavement delineators can better show the forward roadway. Under this scenario, the driver has the best line-of-sight guidance, less mental workload, and the smallest pupil diameter. When only pavement delineators are set, the driver will pay attention to the lamps at the top of the tunnel, so the fixation area will be wider. However, without delineators, the driver feels nervous, and the pupil diameter is the largest.

## Conclusion

Through a simulator study, 22 participants’ eye movement data were collected. By analyzing these data, this paper explored how delineators affect drivers’ visual characteristics, including fixation area and pupil size. The 22 participants’ subjective evaluations of the delineators were obtained by a survey questionnaire. The conclusions are as follows:

- Setting delineators in tunnels can continuously guide drivers’ vision and attract their attention to the pavement. Compared with setting only pavement delineators, setting both wall delineators and pavement delineators can make drivers focus on the forward road as much as possible.
- As large structures, delineators can provide suitable visual stimulation, which can greatly improve drivers’ visual fatigue. Thus, when driving in a tunnel equipped with delineators, drivers exhibit a smaller pupil diameter and lower pupil diameter change rate. In terms of effectiveness, both wall delineators and pavement delineators are recommended. In addition, compared with driving on the left curve and right curve, when driving on the straight section, drivers had the smallest pupil diameter and lowest pupil diameter change rate.
- According to the subjective questionnaire, most participants stated that delineators can delay the appearance of visual fatigue and that setting both wall and pavement delineators is better than setting only pavement delineators. Moreover, almost all participants thought that delineators, especially wall and pavement delineators together, guided their vision very well. The subjective evaluation further confirms the conclusion drawn from the simulation experiment data.

In summary, this paper discusses the influence of delineators on drivers’ vision in tunnels. The results indicate that delineators can greatly improve drivers’ visual conditions and ensure driving safety. However, our experimental design did not consider drivers’ visual characteristics at different speeds in tunnels equipped with delineators. In future research, we will improve this aspect of the design.
